# Age-Related Changes in Acute Phase Reaction, Cortisol, and Haematological Parameters in Ewes in the Periparturient Period

**DOI:** 10.3390/ani11123459

**Published:** 2021-12-05

**Authors:** Monika Greguła-Kania, Urszula Kosior-Korzecka, Agata Hahaj-Siembida, Konrad Kania, Natalia Szysiak, Andrzej Junkuszew

**Affiliations:** 1 Department of Animal Breeding and Agricultural Advisory, Faculty of Animal Sciences and Bioeconomy, University of Life Sciences in Lublin, Akademicka 13, 20-950 Lublin, Poland; gregulakania@gmail.com (M.G.-K.); agata.hahaj-siembida@up.lublin.pl (A.H.-S.); andrzej.junkuszew@up.lublin.pl (A.J.); 2 Sub-Department of Pathophysiology, Department of Preclinical Veterinary Sciences, Faculty of Veterinary Medicine, University of Life Sciences in Lublin, Akademicka 12, 20-033 Lublin, Poland; natalia.szysiak@up.lublin.pl; 3 Department of BioPhysics, Faculty of Environmental Biology, University of Life Sciences in Lublin, 20-033 Lublin, Poland; konrad.kania@up.lublin.pl

**Keywords:** age, cortisol, acute phase proteins, haematological parameters, ewes

## Abstract

**Simple Summary:**

The acute phase response (APR), which comprises a series of specific physiological reactions, is a systemic reaction of the organism to disturbances in its homeostasis caused by infection, inflammation, tissue damage, and stress. Even in healthy ewes, during pregnancy and the transition period, corticosteroids are released which cause the physiological acute phase response. Both in humans and animals, the immune system, like many other physiological systems, is dysregulated with age and a process known as immunoaging occurs. Knowledge of APPs, cortisol, and haematological parameters and factors that influence their alteration could be useful for establishing herd health in ewes during the periparturient period. Understanding how these factors interact with the immune system will help in developing disease control and management strategies that will aid in maintaining good health in ewes and lambs, resulting in greater reproduction.

**Abstract:**

A well-functioning immune system is the basis for protection against infectious and metabolic diseases, and a smooth return to homeostasis. The periparturient period is considered critical because major changes in the endocrine, behavioural, digestive, and immune systems dysregulate immune function, leading to immunosuppression. With age, the immune system could become dysregulated. The purpose of the present investigation was to compare changes in plasma concentrations of acute phase proteins, cortisol, and haematological parameters in the peripheral blood of two age-related groups of healthy ewes to get a better understanding of changes around lambing. Two groups of ewes were enrolled in the study: 3-year-old (young; *n* = 9) and 7-year-old ewes (old; *n* = 9). All females were synchronised and inseminated. In blood plasma, serum amyloid A (SAA) and cortisol concentrations were measured using ELISA tests, a spectrophotometric method to determine haptoglobin (Hp), and a thrombin clottable estimation to determine the fibrinogen (Fb) concentration. The blood parameters were examined using an automated haematological analyser. In clinically healthy ewes, no significant effect of age was observed in SAA, Hp, Fb and cortisol concentration in most of analysed terms. SAA, Hp, Fb, and cortisol fluctuations typical for the periparturient period were observed. There were no age-associated differences in red or white blood cell parameters.

## 1. Introduction

Both in humans and animals, the immune system, like many other physiological systems, is dysregulated with age, and a process known as immunoaging occurs. The changes may be the result of an imbalance in the distribution and a reduction in the number of newly formed T lymphocytes, the main cellular modulators of the acquired immune response, which is caused by the gradual involution of the thymus with age [1,2]. T lymphocytes produce cytokine IL-6, which can also co-stimulate their activation, thus promoting inflammation and increasing the immune response to pathogens [3]. In addition, IL-6 is a major mediator of the induction of acute phase reactions that restore the body’s homeostatic state after infection, inflammation, wounds, or other stress factors. With age, both in humans and animals, the inflammatory activity in the blood increases, and there is an increase in the concentration of TNF-α and IL-6, which also leads to a change in the concentration of acute phase proteins [4]. Franceschi et al. [5] linked a consistently elevated level of cytokines in the elderly to the ‘network theory of aging’. According to the network theory of aging, with age, the defence mechanisms of cells are impaired, and one broken factor that breaks the network of connections leads to a cascade of effects manifested by inflammation. Subclinical chronic inflammaging, along with a weakened, ineffective defence against infectious agents, characterises the impaired function of the immune system.

In addition, age and oxidative stress are believed to be the main causes of hypothalamic cell damage, including dysregulation of the secretory activity of corticoliberin-producing (CRH) hormones [6]. Anterior pituitary dysfunction may affect systemic immune function due to the production of the adrenocorticotropic hormone (ACTH), which stimulates the adrenal cortex to produce corticosteroids. Corticosteroids have a permissive effect on the acute phase reaction process [7]. Additionally, cortisol can enhance the expression of IL-6 receptors in liver cells, thus promoting IL-6-mediated synthesis of APPs. The association of a weakened immune response with age and elevated levels of cytokines in animals has been demonstrated in studies on mice [8] and horses [9].

A well-functioning immune system is the basis for protection against infectious and metabolic diseases, and a smooth return to homeostasis. Additionally, the perinatal period, although it is a physiological state, is associated with a disturbance in homeostasis. The periparturient period is considered critical for ruminants because there are many exogenous and endogenous changes that affect how the immune system responds. Moreover, major changes in the endocrine, behavioural, digestive, and immune systems dysregulate immune function and lead to immunosuppression. The cause of the impaired condition of the immune system in the perinatal period is the change in the leukocyte population, among others. The number of lymphocytes and neutrophils during pregnancy and in the perinatal period fluctuates. Previous studies confirmed that during the perinatal period, there is a reduction in leukocytes compared to the period before pregnancy. Their proportion also changes as lymphocytes decrease, while the number of granulocytes increases [10,11,12]. With age, the functions of monocytes and lymphocytes decrease [13]. This is confirmed by the increasing age sensitivity to mastitis and metritis in cows, which is likely influenced by the weakened function of immune system cells, and further exacerbated by immunosuppression in the perinatal period [14]. In the perinatal period, compared to young cows, older cows showed a lower level of B cells and γδ T lymphocytes, which secrete cytokines and participate in the anti-inflammatory response [15]. These changes also affect the function of blood and milk PMN cells, which in heifers show greater phagocytic and bactericidal activity against *Staphylococus aureus* compared to multiparous cows, which may increase the susceptibility of cows to many infections related to age or parturition [16,17]. 

Reference values are the most frequently used tools to assist in the interpretation of laboratory results. To establish reference values for acute phase proteins, cortisol, and haematological parameters in the perinatal period, knowledge of preanalytical factors that may influence the reference values is needed. Their concentration in the blood of ewes is significantly influenced by the date of the perinatal period [10] and foetal number [18].

Although several studies on changes in inflammation in farm animals have been conducted, in the available literature, there is a lack of data on the effect of age on the acute phase response and perinatal haematological parameters in sheep. The perinatal period is also a critical period for the immunity and health of ewes. The purpose of the present investigation was to compare changes in plasma concentrations of acute phase proteins, cortisol, and haematological parameters in the peripheral blood of two age-related groups of healthy ewes to explore physiological and immunological changes around lambing.

## 2. Materials and Methods

### 2.1. Experimental Animals and Sample Collection

This study was carried out with a flock of the prolific-meat line BCP line ewes (37.5% Polish Lowland Sheep, 12.5% Finnsheep, 25% Berichon du Cher, 25% Charolaise) belonging to the University of Life Sciences in Lublin, Poland, following the procedures approved by the Local Ethics Committee for Animal Testing. Eighteen ewes were enrolled in the study. The young ewes were three years of age in the second lambing (*n* = 9; average age 34.3 ± 0.9 months; mean body weight before insemination 69.2 ± 3.6 kg), and the old ewes were seven years of age in the 6th lambing (*n* = 9; average age 83.6 ± 1.05 months; mean body weight before insemination 73.5 ± 4.2 kg). The animals were kept in the same commercial herd, under identical nutritional and environmental conditions. All ewes were managed according to standard clinical examination procedures to examine the clinical condition of animals [19], and routine serological monitoring for the infection of small ruminant lentiviruses was performed. To exclude subclinical conditions, the haematological and biochemical parameters were determined every month. During the experimental period animals did not get any vaccines. Once a year in autumn, ewes were dehelmintisated. All females were synchronised and artificially inseminated according to Greguła-Kania et al. [18]. The efficiency of insemination was 89%.

Blood samples were collected from the jugular vein of ewes 2 weeks before insemination, 14 and 7 days before parturition, on the day of parturition, and 7 and 14 days after parturition and processed within 4 h for further analysis. Since all females were synchronised and artificially inseminated, lambing took place within three days. Animals were considered healthy if they had an absence of clinical disease during the entire experimental period and if healthy lambs were born. The blood samples were centrifuged at 3000 rpm (603× *g*) for 15 min in a laboratory centrifuge (MPW-350R; MPW Medical Instruments, Warsaw, Poland) at 4 °C. Samples were frozen (−20 °C) prior to subsequent analyses. 

### 2.2. Laboratory Analyses

Plasma serum amyloid A (SAA), haptoglobin (Hp), fibrinogen (Fb) and cortisol concentrations were measured according to Greguła-Kania et al. [18]. 

The blood parameters were determined with colorimetric methods according to the manufacturer’s protocol using reagent kits (BioMaxima, Lublin, Poland; Hydrex Diagnostics, Warsaw, Poland). The complete blood counts were examined using an automated haematological analyser (Abacus Junior Vet, Diatron, Hungary). Each sample was measured in duplicate.

### 2.3. Statistical Analysis

Statistical analysis of the data was performed using the software package Statistica version 13.1. Two-way analysis of variance (ANOVA) was used for repeated measurements to assess the effects of sampling time, age (young ewes vs. old ewes), and their interaction on the measured parameters. The Levene’s test was used to test homogeneity of variance. When the effect of the factors was significant (*p* < 0.05), a post-hoc Tukey test was performed to determine the specific differences between the means.

## 3. Results

The plasma concentrations of APPs and cortisol were significantly higher (*p* < 0.05) in pregnant ewes than before insemination in both analysed groups (Table 1). The SAA increased in the second week before parturition and remained elevated until the end of the study in both groups of ewes (*p* < 0.05). There were no age-associated differences in SAA concentration.

**Figure 1:**
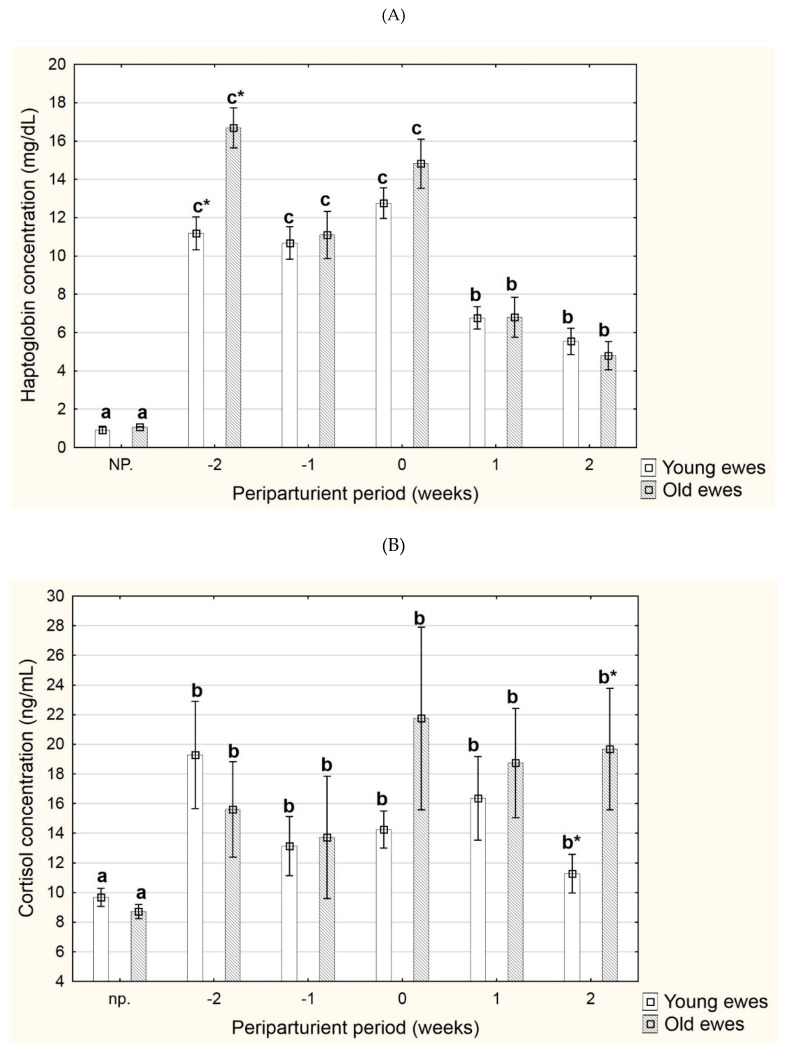
Changes in the concentration of haptoglobin (Hp, mg/dL) (**A**) and cortisol (ng/mL) (**B**) in blood plasma of young and old ewes; Data are expressed as the mean ± standard error (SE); a,b,c indicate that the values obtained at various time points (np., −2, −1, 0, 1, or 2) and denoted with different letters are different (*p* < 0.05); Means within a group (young/old) denoted with * are significantly different (*p* < 0.05). Young ewes were three years of age, and old ewes were seven years of age; (*n* = 18); Abbreviations: NP, non-pregnant ewes (2–3 weeks before insemination); −2, 2 weeks before parturition; −1, 1 week before parturition; 0, parturition; 1, 1 week after parturition; 2, 2 weeks after parturition.

The plasma Hp concentration was reduced in the period from two weeks before up to two weeks after delivery (*p* < 0.05) (Table 1; Figure 1A). In most of the analysed times, the plasma Hp concentration was higher in older ewes than younger ewes, but this difference was statistically significant only two weeks before parturition (*p* < 0.05). In the period from the second week before parturition to parturition, the concentration of haptoglobin in the blood plasma ranged from 10.66 ± 0.66 to 12.80 ± 0.74 mg/dL in young sheep, and from 11.03 ± 1.06 to 16.90 ± 1.09 mg/dL in old sheep. 

The Fb concentration was higher during the whole periparturient period compared to the time before pregnancy (*p* < 0.05). There were no age-associated differences in Fb concentration.

The cortisol concentration did not undergo significant changes during the periparturient period, but the lowest concentration in both age-related groups was observed in non-pregnant ewes (*p* < 0.05) (Table 1; Figure 1B). In the group of older ewes, the cortisol concentration was higher than that in younger ewes one week before parturition until the second week after parturition. This difference was statistically significant in the second week after parturition (*p* < 0.05). 

For most haematological parameters, the differences between the periparturient period and the period before pregnancy were observed (Table 2). The red blood cell (RBC) values were significantly lower throughout the periparturient period compared to the time before gestation (*p* < 0.05). Haematocrit (HTC) decreased gradually, and the lowest value was observed two weeks after parturition (*p* < 0.05). For mean corpuscular volume (MCV) and mean corpuscular haemoglobin (MCH), significantly higher mean values were observed during the periparturient period (*p* < 0.05). 

Differences between the periparturient period and the period before pregnancy were observed for all analysed white blood cell parameters (*p* < 0.05; Table 3). The number of leukocytes was significantly lower throughout the periparturient period (*p* < 0.05). The percentage of lymphocytes decreased, while the percentage of granulocytes increased in the periparturient period compared to the period before pregnancy (*p* < 0.05). The opposite trend was observed for granulocytes, the percentage of which increased throughout the periparturient period.

There were no age-associated differences in red or white blood cell parameters (Table 2 and Table 3). 

## 4. Discussion

Parturition with subsequent metabolic challenges constitutes a potentially stressful event. One of the ways an animal can manifest its stress is by activating an acute phase response, mainly by an increased production of acute phase proteins in the liver. Kovac [20] explained that higher values of acute phase proteins could be related to tissue damage, occurring due to the increased myometrial activity during the expulsion of the calf, involution of the uterus, or degeneration and regeneration of the endometrium.

Research has shown a different metabolic and inflammatory response during the transition period between young and old ewes. In both analysed groups of ewes, the acute phase protein fluctuations during the periparturient period were similar to those reported in previous studies at each sampling point [10]. As reported previously in sheep and cows, the highest haptoglobin plasma concentrations were noted at the time of parturition and decreased sharply in the 1st week after parturition [10,21]. The major biological function of Hp is to bind haemoglobin to prevent haemoglobin-mediated renal parenchymal injury. Free Hb in the blood is toxic and has oxidative activity. Hp binds to it, thus preventing the formation of oxygen radicals (stimulated by iron) and reducing the oxidative damage associated with haemolysis, which defines its role as an antioxidant. Moreover, Hp has a bacteriostatic effect because it binds iron, which is one of the essential elements required for bacterial growth. After formulation of the Hp–Hb complex, iron becomes unavailable for bacteria [22]. In younger primiparous and older multiparous cows, the Hp concentration was higher in primiparous serum [23]. Additionally, during the transition period in pigs and sheep, a higher serum Hp concentration was noted in young primiparous serum, compared to older multiparous serum [24,25]. These changes indicate exacerbated proinflammatory changes in young animals during this period. The increased inflammatory process in young animals also confirms the higher level of biomarkers of oxidative stress in younger ewes [25]. 

In contrast, only animals that developed clinical or subclinical diseases had metabolic and hormonal changes correlated to age compared to healthy animals. In our research, there was no effect of age on Hp concentration in most of the analysed terms. In the second week before parturition, younger ewes had a significantly lower Hp concentration compared to older ewes. Among many changes, clinical or subclinical diseases are characterised by an increase in the number of leukocytes [26]. In our study, in both groups of ewes, the white blood cell (WBC) counts, including both lymphocytes and granulocytes, were within the physiological range, and there was also no difference in WBC counts between groups (Table 3). These data confirm that only healthy animals were included in our study. These results are consistent with an earlier study showing that younger pigs had significantly lower Hp concentrations when compared to older ones, and that there is an effect of health status correlated to the age of serum-haptoglobin concentration [27], but this effect was not caused by age alone. In clinically healthy pigs, no significant effect of age was observed, and an effect of age might indicate subclinical infections. 

Similarly, in studies where the concentration of Hp, cortisol, and ceruloplasmin was analysed in cows of different production statuses, haptoglobin concentrations were significantly higher in the group of cows with clinical symptoms of disease; in the case of subclinical symptoms, there were no differences in haptoglobin and ceruloplasmin [26]. Furthermore, the HPA axis was not activated under subclinical conditions [28]. This presumption was confirmed by research conducted on clinically healthy cows where there were no age-related differences in SAA concentration [23]. In our study, there were no differences between age-related groups in SAA and fibrinogen concentration, but the APP concentration increased during the periparturient period compared to the period before pregnancy. Higher concentrations of these proteins, determined in the last phase of pregnancy and after calving, may relate to the changing hormone profile (the influence of oestrogens and progesterone) [10,20]. The physiological role of SAA is the inhibition of lymphocyte proliferation, oxidative reactions in neutrophils, and platelet and thrombocyte aggregation and detoxification of endotoxins [29]. Fibrinogen binds specifically to the CD11/CD18 receptors on the migrated phagocytes’ surface and releases a cascade of intracellular signals that lead to increased phagocytosis, degranulation, and antibody-dependent cellular cytotoxicity and apoptosis [29].

Conversely, the effect of age in heifers and cows was also noted on cytokine expression [30]. Cytokines are tightly controlled and are mainly produced by macrophages, monocytes, T and B lymphocytes, fibroblasts, hepatocytes, and endothelial cells at the site of inflammatory lesions or infections [31]. Heifers had a significantly higher IL-1B expression than cows at the term of parturition, which can stimulate SAA secretion, and after parturition, the IFNγ expression was significantly higher in older multiparous cows, compared to young heifers [30]. There were no differences in the expression of IL-6, which stimulates Hp. IL2 was significantly higher after parturition in older multiparous cows than in primiparous cows. It is involved in the stimulation of T-cell immune memory and is essential for the proliferation and activation of specific cytotoxic T cells. The higher postpartum IL2 expression level in older multiparous cows may be due to the greater likelihood of exposure to foetal antigens during late pregnancy and delivery compared to heifers. 

Young animals experience greater stress at their first calving, which is reflected in the increase in NEFA concentration after calving [32]. In primiparous sheep, higher levels of cortisol were observed after delivery (at 36 and 48 h postpartum) compared to multiparous sheep [33]. It is probably a consequence of longer births and, consequently, greater stress in primiparous ewes. In older multiparous ewes, the cortisol concentration stabilised 24 h after delivery, and there was a significant decrease, while in younger primiparous it remained elevated during this time.

The level of cortisol was significantly higher only in older multiparous cows with clinical conditions of disease compared to younger primiparous cows, especially if there was more than one disease [26]. In our study, only clinically healthy ewes were enrolled, and a higher cortisol concentration was observed in the older ewe group, but the difference was statistically significant at two weeks after parturition. The results of another study also confirmed that age has no effect on cortisol levels in healthy cows [23,34]. In animals with developed metritis, there is a higher level of cortisol than in healthy ones. Metabolic and hormonal changes, a greater degree of negative energy balance, and increased levels of cortisol, oestradiol, and glucagon were detected only in cows that developed clinical symptoms of disease or subclinical disease [21].

In the blood of ewes, all haematological parameters were measured in physiological values during the analysed period [35] (Table 2 and Table 3). For most haematological parameters, differences were observed between the periparturient period and the period before pregnancy [10]. Observed alterations are characteristic of this stage; maternal and foetal cells are reciprocally recognised by each immune system, resulting in foetal maintenance. During the periparturient period, dilution in the erythrocyte population was observed because red blood cell (RBC), haematocrit (HTC), and haemoglobin (HGB) values were significantly lower (*p* < 0.05) than those observed in non-pregnant ewes. This is a physiological response to decreased blood viscosity, to enhanced blood supply to small vessels, and to the newly formed vascular bed in the uterus and maternal placenta [36]. Differences related to age were not observed. 

Older multiparous ewes are more prone to metabolic stress compared to young ewes. A negative energy balance could impair leucocyte function [37]. During periods of stress, changes in leucocyte percentage occur, causing neutrophilia or lymphopenia [38]. In the analysed groups of ewes, the reference values of white blood cells were observed, but in the older group of ewes, a lower percentage of lymphocytes and a higher percentage of granulocytes (slight lymphopenia and slight neutrophilia) were observed, compared to the group of young ewes. In the second week after parturition, when the milk yield in ewes generally increased, these differences were greatest. Similar results were obtained in healthy cows, and age had no effect on total leukocytes, neutrophils, lymphocytes, basophils, or monocytes [23]. Older multiparous cows had a higher percentage of eosinophils than younger primiparous cows. Moreover, compared to primiparous cows, mitogen-activated PBMCs of multiparous cows produced more IFN-γ, activating IL-2, IL-6, and TNFalfa. Haptoglobin has a suppressive effect on lymphocytes; therefore, the higher Hp concentration reported in the older group of ewes could also explain the tendency toward a lower percentage of lymphocytes in these animals [39]. 

In this study, in clinically healthy ewes, no significant effect of age was observed in SAA, Hp, Fb and cortisol concentrations in most of the analysed terms. SAA, Hp, Fb, and cortisol fluctuations typical for the periparturient period were observed. There were no age-associated differences in red or white blood cell parameters. Knowledge of APPs, cortisol, and haematological parameters and factors that influence their alteration could be useful for establishing herd health in ewes during the periparturient period. Understanding how these factors interact with the immune system will help in developing disease control and management strategies that will aid in maintaining good health in ewes and lambs, resulting in greater reproduction.

## Figures and Tables

**Table 1:** Concentration of acute phase proteins and cortisol in blood plasma of ewes (*n* = 18).

		Non-Pregnant	2 Weeks before Parturition	1 Week before Parturition	Parturition	1 Week after Parturition	2 Weeks after Parturition
Serum amyloid A (µg/mL)	Young ewes	0.31 ± 0.03 ^a^	7.58 ± 0.32 ^b^	8.75 ± 0.29 ^b^	8.15 ± 0.43 ^b^	8.94 ± 0.30 ^b^	9.13 ± 0.37 ^b^
	Old ewes	0.37 ± 0.03 ^a^	8.08 ± 0.48 ^b^	8.52 ± 0.34 ^b^	10.09 ± 1.01 ^b^	10.04 ± 0.81 ^b^	9.37 ± 0.79 ^b^
Haptoglobin (mg/dL)	Young ewes	0.90 ± 0.15 ^a^	11.09 ± 0.89 ^c,^*	10.66 ± 0.66 ^c^	12.80 ± 0.74 ^c^	6.70 ± 0.55 ^b^	5.42 ± 0.63 ^b^
	Old ewes	1.09 ± 0.18 ^a^	16.90 ± 1.09 ^c,^*	11.03± 1.06 ^c^	14.73 ± 1.16 ^c^	6.84 ± 1.08 ^b^	4.83 ± 0.61 ^b^
Fibrinogen (g/L)	Young ewes	3.33 ± 0.20 ^a^	6.85 ± 0.4 ^b^	7.42 ± 0.56 ^b^	8.41 ± 0.53 ^b^	8.93 ± 0.48 ^b^	7.54 ± 0.59 ^b^
	Old ewes	2.98 ± 0.16 ^a^	7.57 ± 0.36 ^b^	6.99 ± 0.86 ^b^	10.67 ± 1.80 ^b^	10.12 ± 1.84 ^b^	8.93 ± 0.68 ^b^
Cortisol (ng/mL)	Young ewes	9.72 ± 0.46 ^a^	19.35 ± 4.00 ^b^	13.07 ± 2.19 ^b^	14.22 ± 1.15 ^b^	16.40 ± 2.62 ^b^	11.31 ± 1.10 ^b,^*
	Old ewes	8.82 ± 0.41 ^a^	15.70 ± 3.22 ^b^	13.70 ± 3.38 ^b^	21.70 ± 6.07 ^b^	18.74 ± 4.29 ^b^	19.84 ± 4.07 ^b,^*

Values represent the mean ± standard error. Means within each row with different superscript letters are significantly different (*p* < 0.05). Means within a group (young/old) denoted with * are significantly different (*p* < 0.05). Young ewes were three years of age, and old ewes were seven years of age.

**Table 2:** Red blood cell parameters in the blood plasma of ewes (*n* = 18).

		Non-Pregnant	2 Weeks before Parturition	1 Week before Parturition	Parturition	1 Week after Parturition	2 Weeks after Parturition
RBC (10^6^/µL)	Young ewes	13.00 ± 0.37 ^b^	8.68 ± 0.38 ^a^	8.78 ± 0.31 ^a^	9.27 ± 0.38 ^a^	9.18 ± 0.48 ^a^	8.69 ± 0.41 ^a^
	Old ewes	12.12 ± 0.93 ^b^	8.93 ± 0.27 ^a^	9.09 ± 0.29 ^a^	8.76 ± 0.42 ^a^	9.18 ± 0.35 ^a^	8.82 ± 0.24 ^a^
HGB (g/dL)	Young ewes	12.68 ± 0.99	11.22 ± 0.48	11.16 ± 0.38	12.50 ± 0.36	12.20 ± 0.57	11.90 ± 0.37
	Old ewes	11.94 ± 1.21	11.14 ± 0.26	11.06 ± 0.48	11.45 ± 0.57	11.75 ± 0.56	11.50 ± 0.52
HCT (%)	Young ewes	33.70 ± 3.58 ^b^	29.87± 1.08 ^ab^	29.98 ± 1.07 ^ab^	32.31 ± 0.93 ^ab^	31.44 ± 1.32 ^ab^	26.63 ± 3.18 ^a^
	Old ewes	33.20 ± 2.96 ^b^	28.90 ± 0.64 ^ab^	29.44 ± 1.01 ^ab^	29.32 ± 1.07 ^ab^	30.33 ± 1.42 ^ab^	26.44 ± 0.91 ^a^
MCH (pg)	Young ewes	9.74 ± 0.85 ^a^	12.94 ± 0.12 ^b^	12.68 ± 0.14 ^b^	13.46 ± 0.27 ^b^	13.29 ± 0.22 ^b^	13.69 ± 0.28 ^b^
	Old ewes	9.50 ± 0.63 ^a^	12.44 ± 0.20 ^b^	12.16 ± 0.21 ^b^	13.12 ± 0.18 ^b^	12.84 ± 0.26 ^b^	13.11 ± 0.41 ^b^
MCV(fl)	Young ewes	26.60 ± 3.16 ^a^	34.40 ± 0.82 ^bc^	34.00 ± 0.57 ^b^	35.00 ± 0.89 ^bc^	34.30 ± 0.63 ^bc^	30.71 ± 0.71 ^b^
	Old ewes	26.40 ± 1.51 ^a^	32.40 ± 0.87 ^bc^	32.40 ± 0.70 ^bc^	33.52 ± 0.83 ^c^	33.14 ± 0.76 ^c^	29.57 ± 0.57 ^ab^
PLT (10^9^/L)	Young ewes	440.7 ± 78.6	391.8 ± 59.6	342.4 ± 86.6	456.0 ± 62.5	306.7 ± 74.4	471.2 ± 68.7
	Old ewes	352.0 ± 38.2	220.8 ± 65.4	354.6 ± 24.90	387.0 ± 65.7	314.5 ± 88.4	489.6 ± 46.5

Values represent the mean ± standard error. Means within each row with different subscript letters are significantly different (*p* < 0.05); Young ewes were three years of age, and old ewes were seven years of age.

**Table 3:** White blood cell parameters in the blood plasma of ewes (*n* = 18).

		Non-Pregnant	2 Weeks before Parturition	1 Week before Parturition	Parturition	1 Week after Parturition	2 Weeks after Parturition
WBC (10^3^/µL)	Young ewes	12.45 ± 1.83 ^b^	4.43 ± 0.54 ^a^	7.97 ± 0.34 ^a^	7.04 ± 0.91 ^a^	7.05 ± 1.01 ^a^	8.73 ± 0.63 ^a^
	Old ewes	10.16 ± 1.49	7.00 ± 0.61	8.03 ± 0.91	9.33 ± 1.08	9.20 ± 0.88	7.94 ± 0.35
LY%	Young ewes	57.92 ± 12.20	48.38 ± 3.44	47.80 ± 2.40	50.70 ± 2.92	53.00 ± 4.04	48.60 ± 3.86
	Old ewes	71.06 ± 13.83 ^b^	49.87 ± 4.03 ^a^	49.92 ± 4.25 ^a^	51.70 ± 3.66 ^ab^	49.87 ± 4.10 ^a^	57.32 ± 2.70 ^a^
GR%	Young ewes	37.68 ± 10.79	51.12 ± 3.41	51.70 ± 2.40	48.80 ± 1.92	46.49 ± 4.04	50.88 ± 4.86
	Old ewes	27.32 ± 12.89 ^a^	49.42 ± 4.04 ^b^	49.57 ± 4.25 ^b^	47.79 ± 3.66 ^b^	49.62 ± 4.10 ^b^	42.17 ± 2.70 ^ab^

Values represent the mean ± standard error. Means within each row with different superscript letters are significantly different (*p* < 0.05). Young ewes were three years of age, and old ewes were seven years of age.

## Data Availability

The datasets used and analysed during the current study are available from the corresponding author upon reasonable request.
